# Percutaneous transforaminal endoscopic surgery (PTES) for symptomatic lumbar disc herniation: a surgical technique, outcome, and complications in 209 consecutive cases

**DOI:** 10.1186/s13018-017-0524-0

**Published:** 2017-02-08

**Authors:** Yu-tong Gu, Zhan Cui, Hong-wei Shao, Yun Ye, Ai-qun Gu

**Affiliations:** 1 Department of Orthopaedics, Zhongshan Hospital of Fudan University, Shanghai, 200032 China; 2 Department of Orthopaedics, Zhenjiang Hospital of Traditional Chinese and Western Medicine, Zhenjiang, Jiangsu 212005 China; 3 Department of Orthopaedics, Taizhou Hospital of Traditional Chinese and Western Medicine, Taizhou, Jiangsu 225300 China

**Keywords:** Lumbar disc herniation, Transforaminal, Endoscopic discectomy, Minimally invasive surgery

## Abstract

**Background:**

We designed an easy posterolateral transforaminal endoscopic decompression technique, termed PTES, for radiculopathy secondary to lumbar disc herniation. The purpose of the study is to describe the technique of PTES and evaluate the efficacy and safety for treatment of lumbar disc herniation including primary herniation, reherniation, intracanal herniation, and extracanal herniation and to report outcome and complications.

**Methods:**

PTES was performed to treat 209 cases of intracanal or extracanal herniations with or without extruding or sequestrated fragment, high iliac crest, scoliosis, calcification, or cauda equina syndrome including recurrent herniation after previous surgical intervention at the index level or adjacent disc herniation after decompression and fusion. Preoperative and postoperative leg pain was evaluated using the 10-point visual analog scale (VAS) and the results were determined to be excellent, good, fair, or poor according to the MacNab classification at 2-year follow-up.

**Results:**

The patients were followed for an average of 26.3 ± 2.3 months. The VAS score of leg pain significantly dropped from 9 (6–10) before operation to 1 (0–3) (*P* < 0.001) immediately after the operation and to 0 (0–3) (*P* < 0.001) 2 years after operation. At 2-year follow-up, 95.7% (200/209) of the patients showed excellent or good outcomes, 2.9% (6/209) fair and 1.4% (3/209) poor. No patients had any form of permanent iatrogenic nerve damage and a major complication, although there were one case of infection and one case of recurrence.

**Conclusions:**

PTES for lumbar disc herniation is an effective and safe method with simple orientation, easy puncture, reduced steps, and little X-ray exposure, which can be applied in almost all kinds of lumbar disc herniation, including L5/S1 level with high iliac crest, herniation with scoliosis or calcification, recurrent herniation, and adjacent disc herniation after decompression and fusion. The learning curve is no longer steep for surgeons.

## Background

The radicular syndrome caused by lumbar disc herniation compressing neurologic elements is a clear indication for surgical decompression. In the last decades, as a minimally invasive surgical technique, the posterior lateral transforaminal endoscopic surgery has been developed to perform discectomy for neurologic decompression under direct view and local anesthesia including YESS (Yeung Endoscopy Spine Surgery) [1–5] and TESS (Transforaminal Endoscopic Spine Surgery) [6–11]. There was a high percentage of patient satisfaction and a low rate of complications in YESS or TESS for lumbar disc herniation [1–11]. Compared with traditional lumbar discectomy, YESS and TESS have certain advantages: (1) no need for general anesthesia, (2) less cases of iatrogenic neurologic damage, (3) no retraction on the intracanal nerve elements, (4) significantly less infections, (5) only minimal disturbance of ligamentum flavum or intracanal capsular structures, therefore, less scar formation, (6) no interference of scar tissue to reach the recurrent herniated tissue in cases of previous dorsal-discectomy, and (7) shorter hospital stay, earlier functional recovery, earlier return to work, and higher cost-effectiveness [1–11]. Although nearly all kinds of disc herniations are accessible for TESS of outside disc-inside technique directly into the spinal canal [2, 3], complexity of C-arm guided orientation, difficulty to find the optimal trajectory for target, and more steps of surgical manipulation leaded to much exposure of X-ray, long duration of operation, and steep learning curve.

We designed an easy posterolateral transforaminal endoscopic decompression technique for radiculopathy secondary to lumbar disc herniation, termed PTES (percutaneous transforaminal endoscopic surgery). The purpose of the study is to describe the technique of PTES and evaluate the efficacy and safety for treatment of lumbar disc herniation including primary herniation, reherniation, intracanal herniation, and extracanal herniation and to report outcome and complications.

## Methods

The clinical study proposal was approved by Zhongshan Hospital Ethical Committee (the medical ethical committee of the authors’ hospital). Informed consent to participate in the study has been obtained from patients. From January 2012 to June 2013, PTES was performed to treat 209 consecutive patients of intracanal or extracanal herniations with or without extruding or sequestrated fragment, high iliac crest, scoliosis, calcification, or cauda equina syndrome including recurrent herniation after previous surgical intervention at the index level or adjacent disc herniation after decompression and fusion. During the same period, other additional patients underwent PTES for various other conditions that did not meet the inclusion criteria for this study. The excluded patients had the primary diagnoses of chronic discogenic pain, foraminal stenosis, lateral recess stenosis, or pyogenic discitis.

Inclusion criteria were as follows: (1) primarily radicular pain of the unilateral leg; (2) disc herniation at one level from L2 to S1 (Table 1) proven by magnetic resonance imaging (MRI) or computed tomography (CT) corresponding to the neurologic findings; (3) clear nerve root tension sign with a straight leg raising sign of less than 45°, or a positive neurologic finding in terms of absent knee or ankle reflex, corresponding dermatomal numbness or weakness of quadriceps, foot/toe extensors, or triceps; and (4) in all patients, conservative treatment had failed.

**Table 1:** Location of lumbar disc herniation according to level

Herniation level	No. of patients	Percent
L5/S1	83	39.7
L4/L5	81	38.8
L3/L4	32	15.3
L2/L3	13	6.2
Total	209	100

**Fig. 1:**
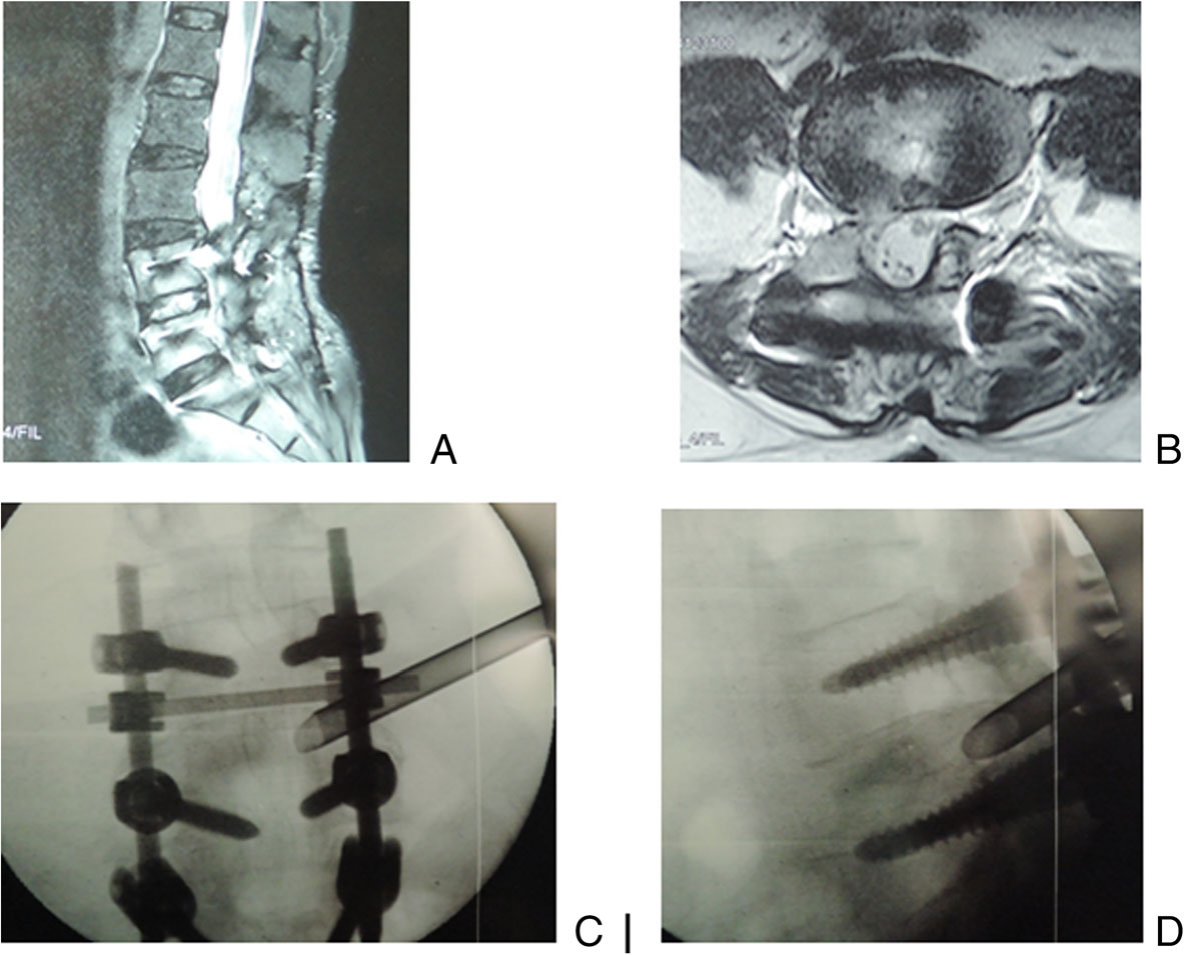
**a** Sagittal and **b** axial MR images showed L4/5 recurrent herniation after decompression and fusion in 61-year-old woman. During the procedure of PTES, **c** posteoanterior and **d** lateral C-arm image confirmed that a 7.5-mm working cannula was advanced directly to the protruding fragment

**Fig. 2:**
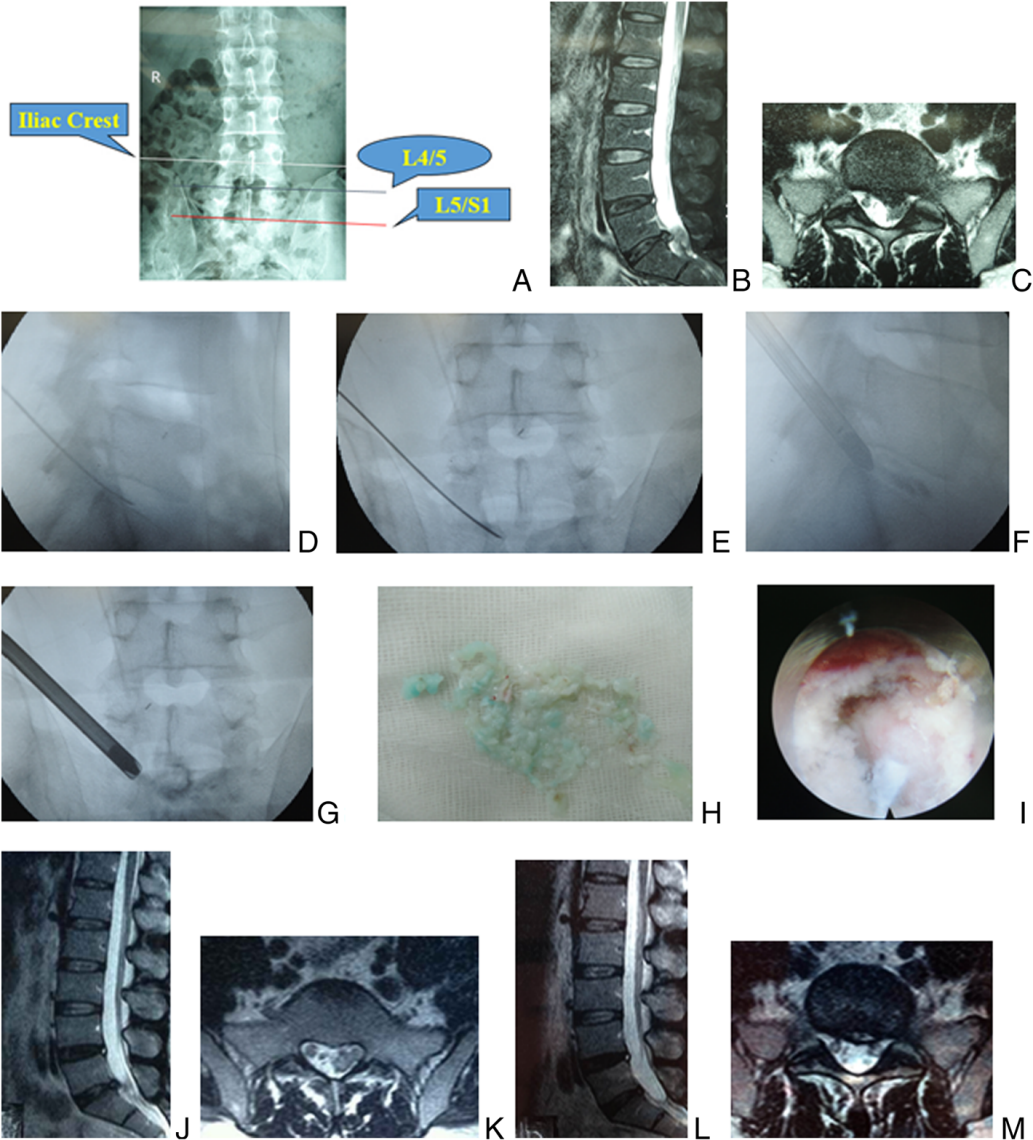
**a** Posteoanterior X-ray picture, **b** sagittal, and **c** axial MR images showed L5/S1 disc herniation with high iliac crest in a 44-year-old man. The tip of the puncture needle was in the posterior one third of intervertebral space on **d** lateral C-arm view and beyond the medial border of pedicle on **e** posteoanterior C-arm view. During the procedure of PTES, a 7.5-mm working cannula was advanced over the guiding rod to the vicinity of the sequestrated fragment on **f** lateral and **g** posteoanterior C-arm view after the enlargement of the foramen. **i** Endoscopic picture showed that the nerve root was exposed for complete decompression after removal of **h** sequestrated disc fragments, which was confirmed on **j** sagittal and **k** axial MR images 1 week after operation. After 2 years follow-up, **l** sagittal and **m** axial MR images showed no recurrent herniation

**Fig. 3:**
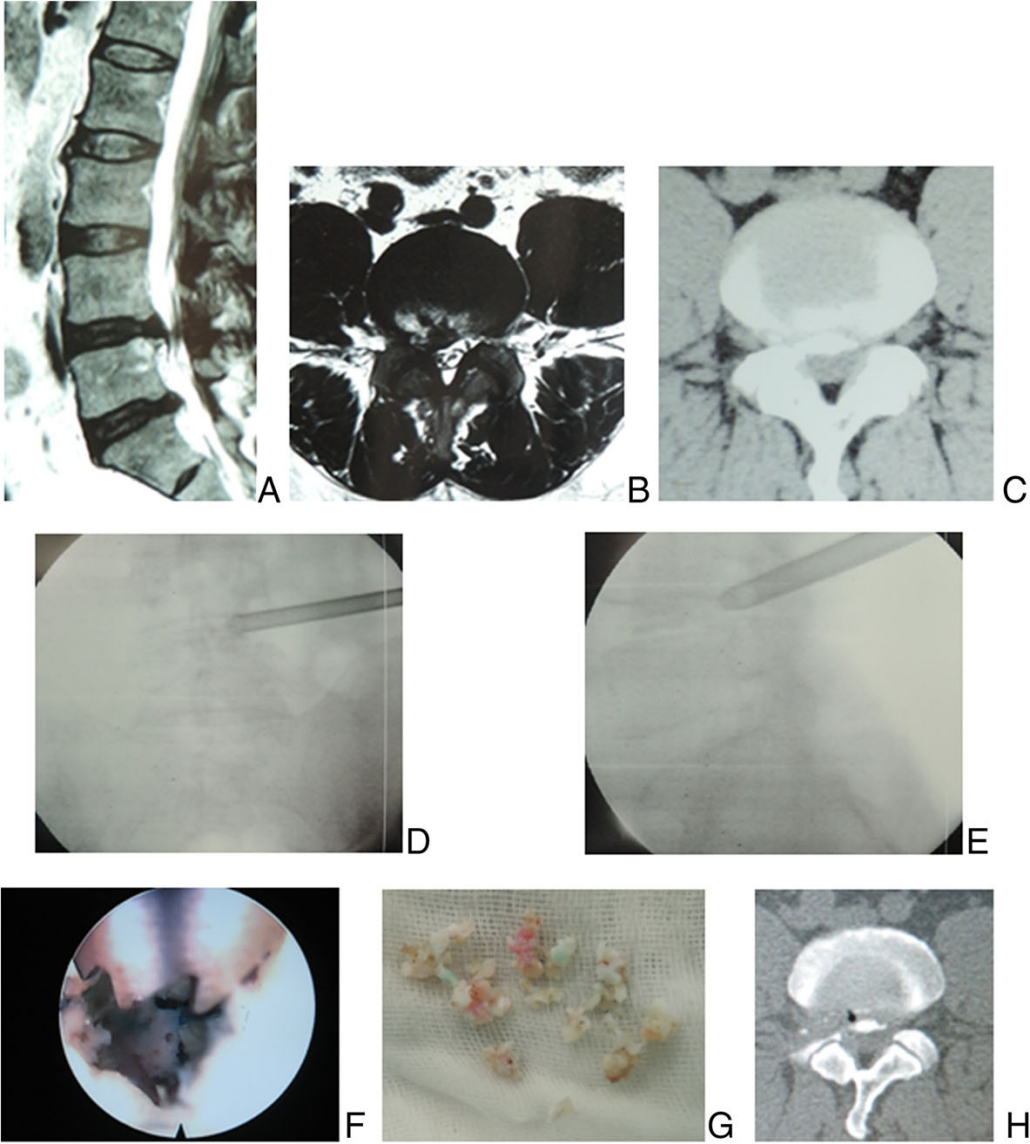
Male patient of 42 years with L4/5 disc herniation and calcification underwent the procedure of PTES. **a** Sagittal, **b** axial MR images, and **c** CT scan showed calcification in L4/5 disc herniation. During the procedure of PTES, **d** posteoanterior and **e** lateral C-arm image confirmed that a 7.5-mm working cannula was advanced directly to the protruding fragment. A 3.5-mm small reamer was used to grind off **g** calcified tissue under **f** view through the endoscope, which was confirmed on **h** CT scan after surgery

This study included 181 intracanal and 28 extracanal (foraminal and extraforaminal) herniations (Table 2). Eighteen recurrent herniations (Fig. 1), three missed fragments after previous surgical intervention at the index level (Table 3), and three adjacent disc herniations after decompression and fusion also were included. For the group that had undergone prior surgical intervention, there were 14 laminectomy and discectomies, 2 endoscopic discectomy, 5 decompression and fusion, 2 radiofrequency ablation, and 1 ozone injections. This study included 29 L5/S1 herniations with high iliac crest (Fig. 2), 25 with scoliosis, and 31 with calcification (Fig. 3) (Table 4), and cauda equina syndrome occurring in 2 patients.

**Table 2:** Location of the lumbar disc herniation in relation to the pedicle and spinal canal

Location of herniation	No. of patients	Percent
Intracanal	181	86.6
Foraminal	16	7.7
Extraforaminal	12	5.7
Total	209	100

**Table 3:** Prior surgical intervention undertaken at index level

Prior procedure at index level	No. of patients	Percent
Laminectomy and discectomy	14	6.7
Endoscopic discectomy	2	1.0
Decompression and fusion	2	1.0
Radiofrequency ablation	2	1.0
Ozone injections	1	0.5

**Table 4:** Outcome according to the MacNab classification

	All patients	L5/S1 herniations with high iliac crest	Herniations with scoliosis	Herniation with calcification
No. of patients in category	209	29	25	31
Excellent or good *n* (%)	200 (95.7)	29 (100)	25 (100)	30 (96.8)
Fair *n* (%)	6 (2.9)	0 (0)	0 (0)	1 (3.2)
Poor *n* (%)	3 (1.4)	0 (0)	0 (0)	0 (0)

### Pre- and postoperative imaging

All patients were evaluated before the procedure by CT and MRI imaging to determine the type of disc herniation (intracanal or extracanal) or to determine if there was calcification. An intracanal herniation is defined as its apex between the bilateral pedicles. The foraminal herniation had its apex within the mediolateral borders of the adjacent pedicle, and the apex is lateral to the bordering pedicle in an extraforaminal herniation. Posteoanterior and lateral radiographs were obtained to detect scoliosis or high iliac crest when the lower plate of L4 vertebral body was not higher than the line between the highest points of bilateral iliac crest (Fig. 2a). After the treatment, MRI images were obtained to assess neurological decompression or exclude dural cyst, myelomeningocele, dural tears or spinal fluid leaks, and reherniation.

### Surgical procedure

Anesthesia consisted of 1% local lidocaine infiltration, supplemented with conscious sedation. The patient was placed in a prone position with hyperkyphotic bolsters placed under the abdomen on a radiolucent table, especially in the cases of L5/S1 herniation with high iliac crest. A biplane fluoroscopy was used for radiograph imaging. Good posteoanterior and lateral images should be obtained by rotating the C-arm relative to the patient whose back was positioned parallel to the horizontal plane especially in the scoliosis case.

**Fig. 4:**
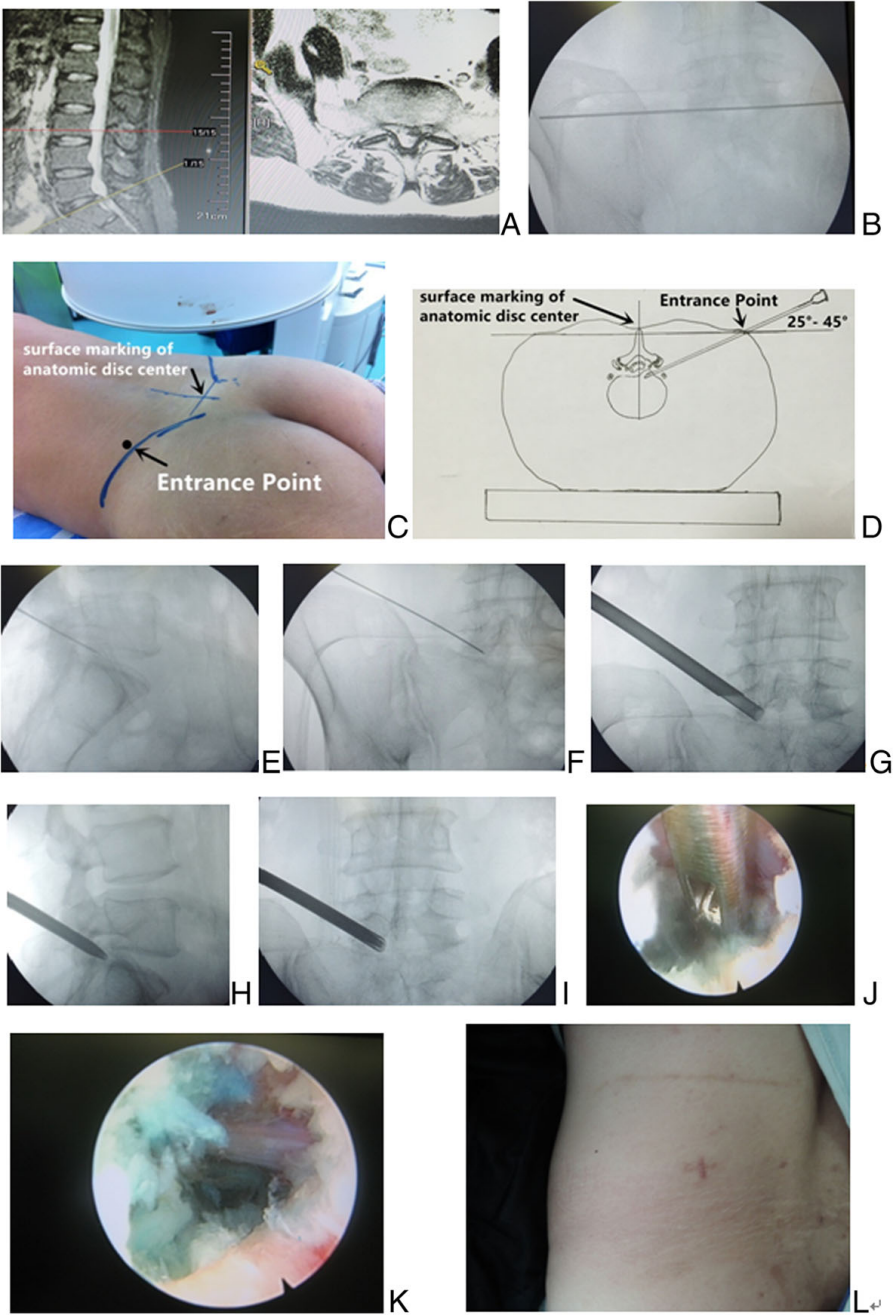
Male patient of 65 years with L5/S1 disc herniation underwent the procedure of PTES. **a** Sagittal and axial MR image showed L5/S1 disc herniation. A transverse line bisecting the disc was drawn along the metal rod which was placed transversely across the center of the target disc on **b** posteoanterior C-arm view. **c** Photography showed the surface marking of anatomic disc center identified by the intersection of transverse line and longitudinal midline, and the entrance point of puncture located at the corner of flat back turning to lateral side. The puncture needle was inserted at about 35° angle (25°–45°) to the horizontal plane anteromedially toward the perpendicular line through anatomic disc center on **d** schematic diagram. The tip of puncture needle was in the posterior intervertebral space close to spinal canal on **e** lateral C-arm view and near the medial border of pedicle on **f** posteoanterior C-arm view. Through the 8.8-mm cannula, a 7.5-mm hand reamer was introduced to ream facet bone until the resistance faded, which was checked with **g** posteoanterior image. **h** Lateral C-arm view showed that the tip of the thick guiding rod was positioned at the herniated fragment. A 7.5-mm working cannula was advanced over the guiding rod directly to the sequestrated fragment on **i** posteoanterior fluoroscopic image. Under **j**, **k** endoscopic view, the fragments underneath the nerve root were removed and the freed nerve root could be visualized. **l** Photography showed minimally invasive result 1 months after surgery

**Fig. 5:**
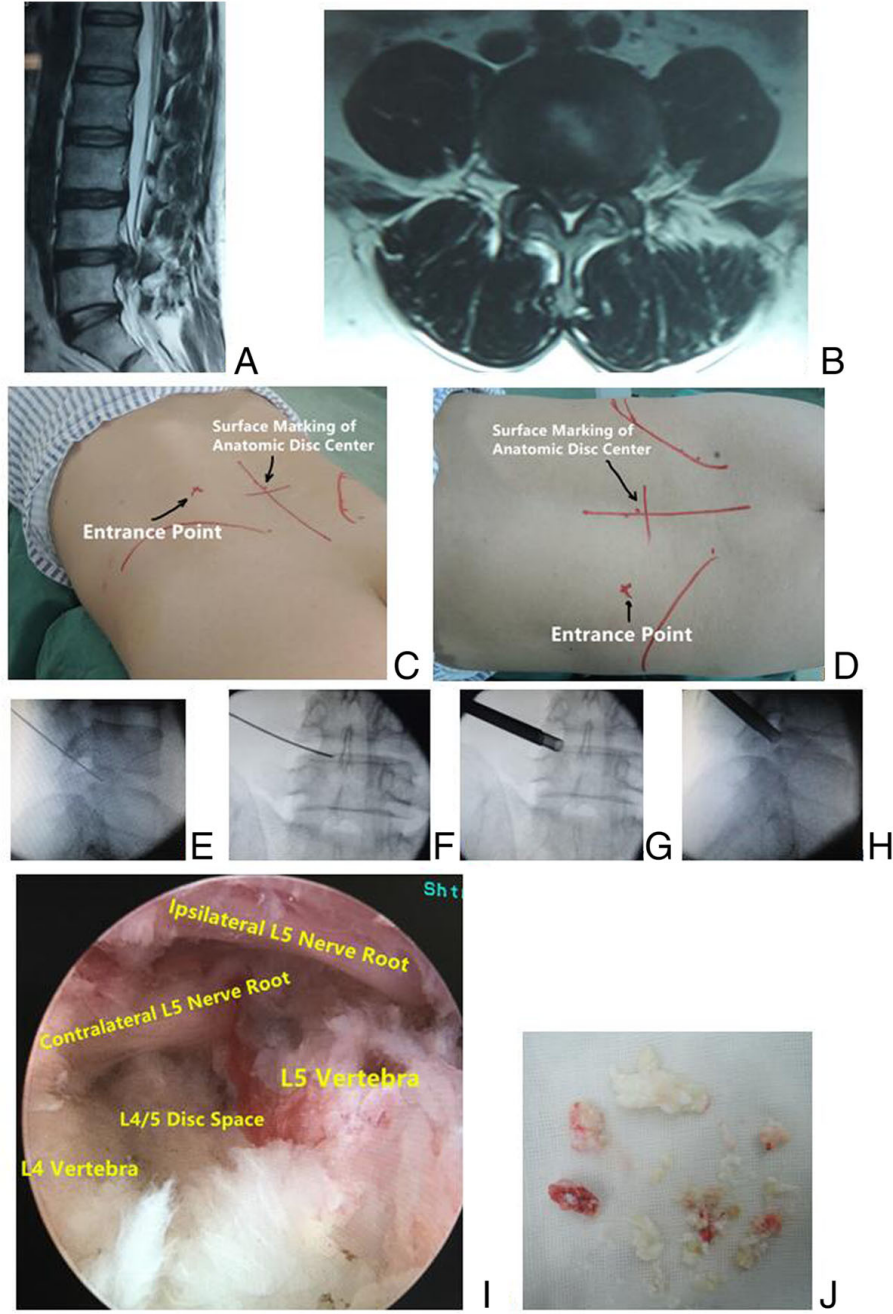
**a** Sagittal and **b** axial MR images showed L4/5 disc herniation in 41-year-old man. **c**, **d** Photography showed the surface marking of anatomic disc center identified by the intersection of transverse L4/5 level line and longitudinal midline, and the entrance point of puncture located at the corner of the flat back turning to the lateral side. The tip of puncture needle was in the posterior one third of intervertebral space on **e** lateral C-arm view and beyond the medial border of pedicle on **f** posteoanterior C-arm view. A 7.5-mm hand reamer was introduced through the cannula to ream away the ventral bone of superior facet joint and ligmentum flavum for enlargement of foramen (press-down enlargement of foramen) until the resistance disappeared. The tip of reamer should exceed the medial border of pedicle till the midline between the pedicle and spinal process on **g** posteoanterior C-arm view, and it should be close to posterior wall of target disc on **h** lateral C-arm view. **i** Endoscopic picture showed that both the ipsilateral nerve root and the contralateral nerve root were exposed for complete decompression after removal of **j** sequestrated disc fragments

The midline is marked on the skin surface by palpating the spinal processes. Then a transverse line bisecting the disc is drawn along the K-wire which is placed transversely across the center of the target disc on the posteoanterior image (Fig. 4b). The surface marking of anatomic disc center is identified by the intersection of transverse line and longitudinal midline (Figs. 4c, d and 5c, d), which is used as the aiming reference point of puncture.

In practice, we found that the entrance point was located at the corner of the flat back turning to the lateral side (Figs. 4c, d and 5c, d), named “Gu’s Point”, not depending on the size of the patient, gender, and level. This point is as high as, or more cranial, or slightly more caudal than the horizontal line of target disc. The entrance point for the patient with scoliosis is adjusted medially or laterally according to the rotation.

**Fig. 6:**
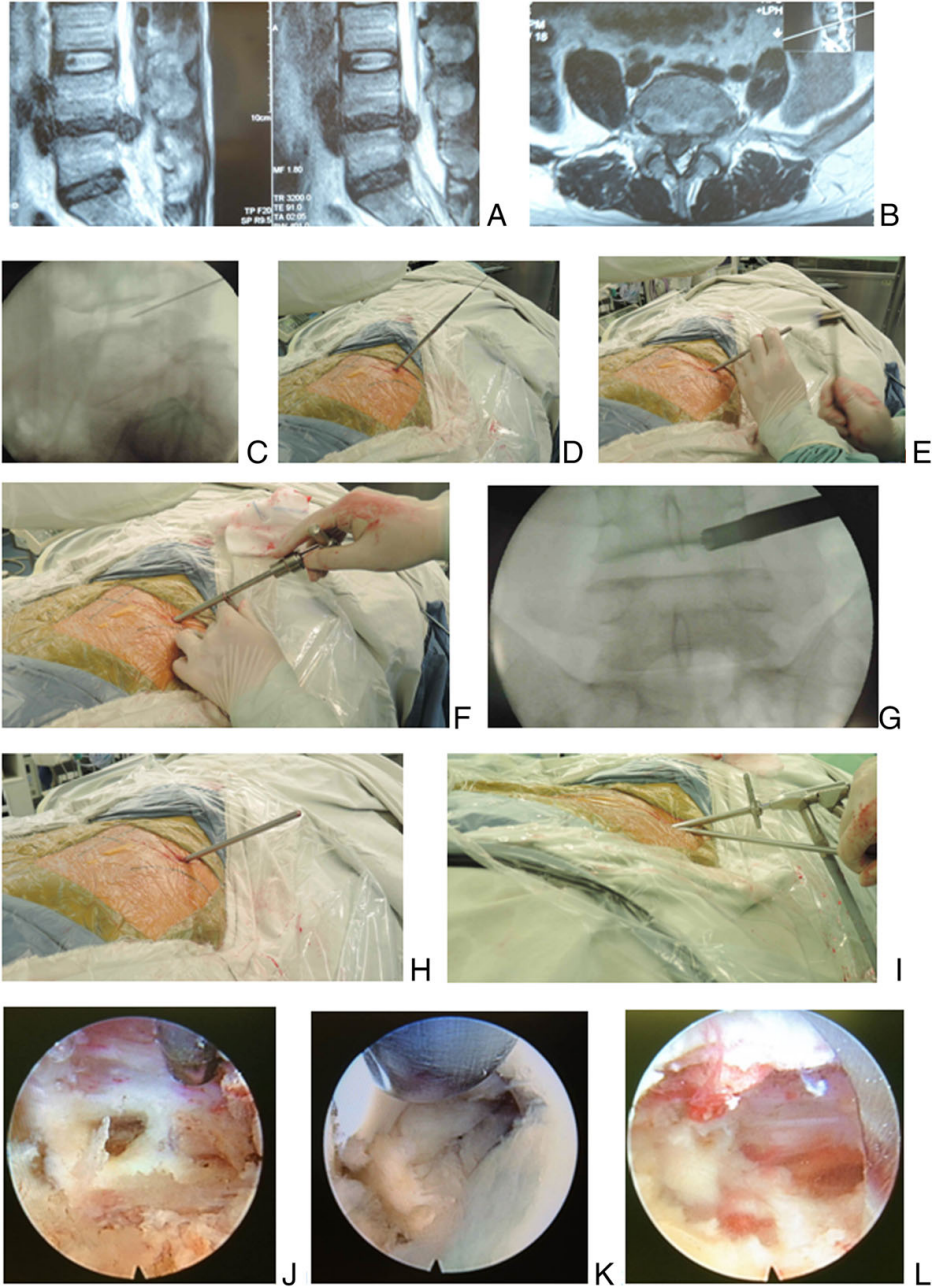
Male patient (47 years) of L4/5 disc herniation with extruding fragment underwent the procedure of PTES. **a** Sagittal and **b** axial MR images showed L4/5 disc herniation. During the procedure of PTES, The puncture needle was anteromedially inserted at about 45° angle to the horizontal plane and the tip of puncture needle was in the posterior one third intervertebral space on **c** lateral C-arm view. Over the guiding wire, stepwise-dilating cannulas were introduced to annulus fibrosus through the foramen **d**. The thick guiding rod was advanced with a mallet into the foramen after removal of the guiding wire **e**. An 8.8-mm cannula was pushed over the rod to the facet joint area, docked at the superior facet after the rod was  removed, and pressed down to make the angle of the cannula to the horizontal plane smaller. Through this cannula, a 7.5-mm hand reamer was then introduced to ream the ventral part of facet bone until the resistance faded (press-down enlargement of the foramen) **f**, which was checked with **g** posteoanterior C-arm image. The reamer was removed, and the thick guiding rod then was reintroduced and advanced with the aid of a mallet until the tip of the guiding rod was into the herniated fragment **h**. A 7.5-mm working cannula was advanced over the guiding rod directly to the extruding fragment and the spine endoscope was inserted **i**. Under **j**, **k**, and **l** endoscopic view, the extruding disc fragment could be observed and the freed nerve root could be visualized after the massive nucleus underneath the nerve root were removed

Then the skin, subcutaneous tissue, and trajectory tract were infiltrated with local anesthesia, and an 18-gauge needle was inserted at about 35° angle (25°–45°) to the horizontal plane anteromedially toward the perpendicular line through the anatomic disc center (Fig. 4d), which should be adjusted according to the rotation of vertebra for scoliosis. Once the needle reached the target when resistance disappeared, the lateral C-arm view was taken to ensure that the tip should be in the posterior one third of the intervertebral space or intracanal area close to the posterior wall of the disc (Figs. 2d, 4e, 5e, and 6c). The plane of the needle bevel could be used for minor directional adjustment. Sometimes the direction of the needle bevel was rotated to skirt the obstacle for target especially at L5/S1 level with high iliac crest. The angle and direction of puncture or the entrance point should be adjusted once there was nerve root symptom. Then the C-arm was rotated to the posteroanterior projection, and the needle tip should be near the medial border of the pedicle on the C-arm view (Figs. 2e, 4f, and 5f). Subsequently, contrast solution [9 ml iohexol (300 mg/mL) + 1 ml methylene blue] or methylene blue solution (9 ml 0.9% NaCl + 1 ml methylene blue) was injected to dye the nucleus, and pain reaction or dye leakage was recorded, which sometimes could be omitted.

**Fig. 7:**
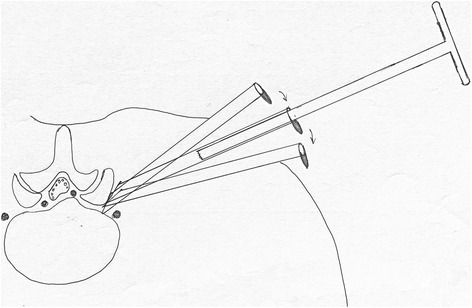
In case the extruding or sequestrated fragment could not be achieved, the step of reaming facet was repeated to remove more ventral parts of the superior facet for further enlargement of the foramen through the cannula docked at the facet and pressed down further to make the angle of the cannula to the horizontal plane smaller

After the guiding wire was introduced into the disc space, a stab incision of about 6 mm was made and the 18-gauge needle was inserted along the guiding wire to the facet joint capsule for lidocaine infiltration. Over the guiding wire, stepwise-dilating cannulas of 1.5, 3.5, and 5.5 mm were introduced to the anulus fibrosus through the foramen (Fig. 6d). After the dilating cannulas were removed, the thick guiding rod of 6.3 mm in diameter was introduced over the guiding wire and then advanced with a mallet into the foramen after removal of guiding wire (Fig. 6e). An 8.8-mm cannula of one-sided opening was pushed over the rod to the facet joint area, docked at the superior facet after the rod was removed, and pressed down to make the angle of cannula to the horizontal plane smaller according to the position of the needle tip on the lateral and posteroanterior C-arm view, which indicates the inclination angle of puncture trajectory. A 7.5-mm hand reamer was then introduced through this cannula to ream away the ventral bone of the superior facet joint and the ligmentum flavum for enlargement of foramen until resistance disappeared, meaning that the spinal canal has been entered, which was called press-down enlargement of foramen (Fig. 6f). The tip of the reamer should exceed the medial border of the pedicle till the midline is between the pedicle and the spinal process on a posteoanterior C-arm view (Figs. 4g, 5g and 6g), and it should be close to the posterior wall of the target disc on the lateral C-arm view (Fig. 5h), to ensure that the vicinity of the extruding or sequestrated fragment was reached. Only the posteoanterior C-arm image was needed to determine the position of the reamer during the foramen enlargement and it could be evaluated through endoscopic image replacing lateral C-arm image whether the target area had been reached. In case this could not be achieved, the step of reaming facet was repeated to remove more ventral parts of the superior facet with 7.5-mm reamer for further enlargement of foramen through the 8.8-mm cannula docked at the facet and pressed down further to make the angle of cannula to the horizontal plane smaller (Fig. 7). In cases with difficult access to the fragment, particularly in the presence of foraminal stenosis, an 8.8-mm reamer was used to enlarge the foramen through a 10-mm cannula. The reamer could be advanced deeper to remove some calcified tissue of disc for lumbar disc herniation with calcification. If the reamer in the foramen was higher or lower than the disc level on the posteoranterior C-arm image, the position and direction of the reamer could be adjusted through a minor movement of the cannula or a thick guiding rod. Once there was nerve root symptom, the surgeon must stop reaming immediately, adjust the angle and direction of reamer (move the tip of cannula laterally and dorsally toward the superior facet and press the cannula down), or change the entrance point medially until the symptom disappear. The reamer was removed, and the thick guiding rod was reintroduced and advanced with the aid of a mallet till the tip of the guiding rod was into the herniated fragment (Fig. 6h). A 7.5-mm working cannula with a one-sided opening was then advanced over the guiding rod directly to the target area (Figs. 1c, d, 2g, 3d, e, 4i, and 6i). For extracanal disc herniation, the process of reaming could usually be omitted.

The spine endoscope was introduced (Fig. 6i), and generally, the extruding or sequestrated disc fragment could be observed and the nerve root was then shown on the screen (Figs. 2i and 5i) after the fragments were removed. In cases where the nerve root was also visible, a working forceps was introduced through the endoscope to remove the fragments underneath the nerve root and the central dura, even the contralateral nerve root, under endoscopic view (Figs. 4j, 5i, and 6j, k). In disc protrusion where the annular wall was complete, the annulus was perforated to pull out the herniated nucleus. The massive disc fragment could be grabbed and extracted together with the endoscope. After the scope was again inserted, the nerve root was inspected (Figs. 4k, 5i, and 6l) and the remaining fragments of the disc were removed under endoscopic vision. When the calcification was encountered, a 3.5-mm small reamer or electric shaver was used to grind off the calcified tissue under the view through the endoscope (Fig. 3f). The cannula was then rotated cephalad to check the existing nerve root and rotated back to detect bone fragments that reamed off and take those out. The freed nerve root that was always pulsating with the heart rate could be observed at the end of procedure (Figs. 2i, 4k, 5i, and 6l). The trigger-flex bipolar radiofrequency probe could be used to clean the access way, stop the bleeding, ablate the nucleus and annulus, shrink the annular tears, and release the scar tissue around the nerve root for recurrent herniation after previous surgical intervention at the index level.

The patients were mobilized 5 h after surgery. A flexible back brace was used for 3 weeks. After leaving the hospital, patients were encouraged to resume their daily routine and were followed up as outpatients at the hospital ward.

### Clinical follow-up

Leg pain was evaluated using the 10-point visual analog scale (VAS) preoperatively, immediately, 1 week, 1, 2, 3, and 6 months, and 1 and 2 years after surgery. The clinical evaluation included a straight leg raising test and a check of the strength of the quadriceps, foot/toe extensors, as well as triceps strength.

In a retrospective assessment, the results were determined to be excellent, good, fair, or poor according to the MacNab classification [12] (Table 5) 2 years postoperatively. The fair and better grades also included the requirement that the patients were willing to select the same procedure again for the same problem in the future.

**Table 5:** MacNab classification [12]

Results	Complications
Excellent	No pain; no restriction of activity
Good	Occasional back or leg pain not interfering with the patient’s ability to do his or her normal work, or to enjoy leisure activities
Fair	Improved functional capacity, but handicapped by intermittent pain of sufficient severity tocurtail or modify work or leisure activities
Poor	No improvement or insufficient improvement toenable an increase in activities/or furtheroperative intervention required

During the follow-up, all complications were registered including iatrogenic nerve damage, vascular injuries, infection, wound healing, thrombosis, or recurrence.

### Statistical analysis

Comparison of preoperative and postoperative VAS was performed using linear mixed effects model for multiple comparison procedures. Statistically significant differences were defined at a 95% confidence level. The SPSS software (17.0, SPSS Inc., Chicago, IL, USA) supported statistical evaluation.

## Results

The 209 patients who met the inclusion criteria were followed for an average of 26.3 ± 2.3 months. There were 116 (55.5%) male patients and 93 (44.5%) female patients. The average ages were 46.4 ± 14.9 years for the male patients and 46.8 ± 11.1 for the female patients.

The mean duration of the operation was 50.9 ± 9.9 min per level. The mean frequency of intraoperative fluoroscopy was 5 (3–14) times per level. The mean blood loss was 5 (2–20) ml per level. The mean stay in the hospital was 3 (2–4) days.

The VAS score of leg pain significantly dropped from 9 (6–10) before operation to 1 (0–3) (*P* < 0.001) immediately after the operation and to 0 (0–3) (*P* < 0.001) 2 years after operation. However, there were 16 (7.7%) patients with the rebound effect of leg pain, and 9 (7–10) of VAS score preoperatively dropped to 0 (0–2) immediately after operation and rose to 7 (5–9) 1 week postoperatively. Fourteen of 16 patients got pain relief during 2 months, and the VAS score became 3 (2–4) 2 months postoperatively and 0.5 (0–3) 2 years postoperatively. Other two underwent reoperation about 1 month after surgery in other hospitals.

At 2-year follow-up, 95.7% (200/209) of the patients showed excellent or good outcomes, 2.9% (6/209) fair, and 1.4% (3/209) poor. The percentages of excellent or good results were 100% (29/29) for L5/S1 herniations with high iliac crest, 100% (25/25) for herniations with scoliosis, and 96.8% (30/31) for herniation with calcification. The percentage of fair results was 3.2% (1/31) for herniation with calcification (Table 4). Excellent or good outcome were shown in 100% (24/24) of recurrent herniations or missed fragments after previous surgical intervention at the index level and adjacent disc herniations after decompression and fusion. Voiding dysfunction recovered during 1 day after surgery and the strength of the quadriceps, foot/toe extensors, or triceps improved during 3 months in two patients of cauda equine syndromes showing fair outcomes.

Postoperative complications (Table 6) included three patients who experienced increased weakness of quadriceps or foot/toe extensor strength, which fully recovered about 1 month after surgery. One other patient experienced low toxicity infection of disc, which was cured by intravenous use of antibiotics for 2 weeks. There was one case of herniation recurrence 8 months after surgery, which was successfully treated with MIS-TLIF (minimally invasive surgery-transforaminal lumbar intervertebral fusion). No dural tears had to be treated and no dural leaks after surgery occurred, and no meningoceles or dural cysts in the surgical area were observed in the postoperative MRI scans. No patients had any form of permanent iatrogenic nerve damage and a major complication such as intraoperative vascular injury. There was no death.

**Table 6:** Complications

Complication	Number	Percent
Increased weakness of quadriceps or foot/toe extensor strength	3	1.4
Disc infection	1	0.5
Herniation recurrence	1	0.5
Permanent iatrogenic nerve damage	0	
Dural tear or dural leak	0	
Intraoperative vascular injury	0	
Death	0	

## Discussion

In 2002, Yeung et al. [5] reported the outcome and complications in 307 cases of posterolateral endoscopic discectomies (YESS) with a minimal follow-up of 1 year. They reported an 83.6% excellent or good result and a 9.3% rate of poor results. Their reoperation rate was 5%, with an average follow-up of 19 months. In 2006, the study by Hoogland et al. [10] showed that there was a recurrence rate of 6.9% at 1-year postoperatively in 142 cases treated with posterior lateral endoscopic discectomy (TESS) and at 2-year follow-up, 85.4% of the patients had an excellent or good result and 7.7% were not satisfied. These are comparable to the outcomes in our study of posterolateral endoscopic discectomy (PTES). At 2-year follow-up, 95.7% (200/209) of the patients showed excellent or good results, 2.9% (6/209) fair, and 1.4% (3/209) poor. The recurrence rate was 0.5% (1/209), and the reoperation rate was 1.4% (3/209).

Since 1994, Hoogland et al. [6–9] have used special reamers to enlarge the foramen, which was named after TESS, so that the anterior spinal canal could be made accessible for endoscope and instruments also for the L5/S1 level, avoiding injury to the exiting nerve root, a problem that has been reported after the regular transforaminal approach. At that point, nearly all types of disc herniation became accessible with the lateral percutaneous approach [13]. However, in TESS, it was complicated to determine the entrance point through C-arm-guided orientation, and it depended on the measurement of distance to the midline, which sometimes was not accurate because of the different sizes of patients. The tip of the puncture should reach the posterior wall of the disc or the superior fact in TESS, and the rigid target of puncture made it difficult to find the optimal trajectory through repeated directional adjustments. In addition, there were more steps for enlargement of foramen through step-by-step reaming of the superior facet bone during the procedure. These led to much exposure of X-ray, long duration of operation, and steep learning curve.

For the orientation before puncture in PTES, only the posteroanterior C-arm projection was needed when the K-wire was placed transversely across the center of the target disc. A transverse line bisecting the disc was drawn along the K-wire, and the surface projection of anatomic disc center was located where the transverse line crossed the longitudinal midline drawn by palpation. The puncture should aim at the perpendicular line through the anatomic disc center. Our study showed that the entrance point was located at the corner of the flat back turning to the lateral side, and as high as, or more cranial, or slightly more caudal than the horizontal line of target disc, which was similar to “All roads lead to Rome (herniated fragment).” This has never been mentioned by other scholars, and we named this entrance point after “Gu’s Point”. It was not necessary to take the C-arm projection and measure the distance to the midline for determination of the entrance point. The orientation before puncture in PTES was simplified compared with TESS. On the lateral C-arm view, the target of puncture should be in the posterior one third of intervertebral space or intracanal area close to posterior wall of the disc in PTES, which made the direction and angle of the puncture flexible. Sometimes, the needle was inserted into a disc at 45° angle to the horizontal plane for puncture, which still made it achievable to put the working cannula into the spinal canal (Fig. 6). So, in PTES technique it was easy to find the optimal trajectory of puncture.

Although the tip of the puncture needle was in the disc and sometimes higher or lower than the target disc, the guiding rod of 6.3 mm in diameter could be advanced into the foramen with a mallet after the guiding wire was drawn out, and good position could be more easily achieved by minor adjustment of guiding rod compared with the soft puncture needle. When enlargement of foramen was performed through the cannula docked at the facet during PTES, the cannula was pressed down to make the angle of the reamer to the horizontal plane smaller and more bones in the ventral part of the superior facet was cut away (Fig. 6f). We called it press-down enlargement of foramen, which made it easy to advance the working cannula into the spine canal between the dura and disc even if the angle of puncture was 45° and to remove the fragments underneath the nerve root and the central dura, even the contralateral nerve root (Fig. 5i), without the retraction on the intracanal nerve elements. If the reamer in the foramen was higher or lower than the disc level, the position and direction of the reamer could be adjusted through minor movement of the cannula or a thick guiding rod. If the vicinity of the extruding or sequestrated fragment could not be reached, the following steps were needed: (1) repeated reaming of more bones of the ventral part of the superior facet using a 7.5-mm reamer through the 8.8-mm cannula pressed down further, which could be achieved by moving the tip of the cannula laterally and dorsally toward the superior facet (Fig. 7) or (2) using a bigger reamer of 8.8-mm through a 10-mm cannula for further enlargement of the foramen in the presence of foraminal stenosis. All these measures made it possible to simplify the orientation and facilitate the puncture of PTES.

During PTES, after the orientation and puncture, enlargement of the foramen was performed by one-step reaming of a 7.5-mm reamer instead of a step-by-step reaming before endoscopic discectomy. Simple orientation, easy puncture, and reduced steps could decrease the times of C-arm projection. The results of a study showed that the mean frequency of intraoperative fluoroscopy was 5 (3–14) times per level. In general, 4 times of C-arm projection were needed during the procedure of PTES, including the orientation of the involved disc center on the posteoranterior image (Fig. 4b), confirmation of the puncture needle tip reaching the target on the lateral (Fig. 4e) and posteoranterior view (Fig. 4f), and the position of the reamer checked with an posteoanterior image (Fig. 4g) when resistance faded. It could be ensured by the endoscopic image replacing lateral fluoroscopic projection that the vicinity of the extruding or sequestrated fragment had been reached. In extracanal disc herniation, the procedure of reaming could usually be omitted and there were only three times of intraoperative fluoroscopy during PTES (Fig. 4b, e, f). The amount of radiation, which the surgeon and the patient received, could be reduced as little as possible in the procedure. Compared with TESS, simple orientation, easy puncture, reduced steps, and few C-arm projections shortened the duration of PTES procedure and lowered the learning curve. From positioning the patient to closing the skin, the mean duration of operation was 50.9 ± 9.9 min per level.

L5/S1 herniation with high iliac crest and disc herniation with scoliosis or calcification are difficult cases for transforaminal endoscopic surgery. In our study, the percentages of excellent or good results were 100% (29/29) for L5/S1 herniations with high iliac crest, 100% (25/25) for herniations with scoliosis, and 96.8% (30/31) for herniation with calcification. The percentage of fair results was 3.2% (1/31) for herniation with calcification. The following are some key points to overcome the difficulties for these special cases. In L5/S1 disc herniation with high iliac crest, the puncture needle usually was blocked by iliac crest, sacral promontory or transverse process. The patient should be hyperkyphoticly placed in a prone position in order to make the space larger among the iliac crest, sacral promontory, and transverse process. The obstacle could be skirted through rotating the direction of the needle bevel during the puncture. For disc herniation with scoliosis, the C-arm should be rotated relative to the patient to obtain good posteoanterior and lateral images, and the entrance point and angle of puncture should be adjusted according the rotation of lumbar vertebrae. When there was calcification in disc herniation, some calcified tissue of the disc could be reamed away during enlargement of the foramen, and the small reamer or electric shaver was used to grind off the calcified tissue through the endoscope under the view.

In recurrent herniation after previous surgical intervention at the index level, the lateral transforaminal approach could bypass the scar tissue in the previous dorsal area and reduce the risk of dural tears. Hoogland et al. [11] reported that the spinal fluid leak during the surgery was suspected in 11 cases, and no dural tears had to be treated in their series of 262 patients of recurrent disc herniation undergoing endoscopic transforaminal discectomy. No dural leaks after surgery occurred or meningoceles or dural cysts in the surgical area were observed in the postoperative MRI scans that were obtained on almost all patients. But the incidents of dural tears requiring treatment in the dorsal microdiscectomy is about 10% [14]. In our series of 18 patients treated by PTES, there was no dural tears, and no dural leaks or meningoceles or dural cysts in the surgical area after surgery. The extruding or sequestrated disc material could be removed through the working tunnel of lateral transforaminal approach without interference with scar tissue. After removal of the protruded material, the nerve root could be inspected and the scar tissue around the nerve root could be released by the radiofrequency using a trigger-flex bipolar probe. In comparison, the scar must be removed in the dorsal reintervention, and tedious retraction of the compressed nerve root was needed for the removal of protruded disc tissue, which increased risk of neurologic injury. When PTES was performed to treat recurrence herniation and adjacent disc herniation after decompression and fusion, there was no need to remove, replace, or extend the previous internal fixation, which significantly decreased the aggressiveness, reduced the blood loss, and fastened the recovery, compared with the open revision surgery.

The patient was in a continuous awakened state under local anesthesia supplemented with intravenous sedation during surgery of PTES, and the surgeon could be alerted if there was any physical irritation to the neurologic elements. Once there was nerve root symptom during puncture or enlargement of foramen, which usually indicated the involvement of exiting nerve root, the performance must be stopped immediately. The angle and direction of puncture should be adjusted or the entrance point should be medially moved until the symptom disappeared during puncture. The surgeon could change the angle and direction of the reamer through moving the tip of the cannula laterally and dorsally toward the superior facet and pressing the cannula down to avoid the irritation of an exiting nerve root during the enlargement of the foramen. In case there was still neurologic symptom during the reaming of the superior facet, the entrance point should be medially changed. Although no patient had any form of permanent iatrogenic nerve damage in this study, there were three patients who experienced transient weakness of quadriceps or foot/toe extensor strength, which was relative to no stopping the operation immediately when the nerve root symptom occurred. In addition, there were 16 (7.7%) patients with the rebound effect of leg pain 1 week after operation, in which 14 cases got pain relief during 2 months and other two underwent reoperation after about 1 month in other hospitals. This indicated that the observation of at least 2 months should be preferred to immediate reoperation when the rebound effect of leg pain occurred, although further study should be performed to detect the possible factors. There was only one case of recurrence in this study and 0.5% (1/209) of recurrence rate was significantly lower, compared with that of other reports. Attention should be paid to take good care of the lumbar spine after surgery, such as not bending frequently, no lifting of heavy load, and not keeping the same position for a long time, which was an important factor of preventing recurrent herniation.

## Conclusions

The current data indicate that PTES for lumbar disc herniation is an effective and safe method with simple orientation, easy puncture, reduced steps, and little X-ray exposure, which can be applied in almost all kinds of lumbar disc herniation, including L5/S1 level with high iliac crest, herniation with scoliosis or calcification, recurrent herniation, and adjacent disc herniation after decompression and fusion. The learning curve is no longer steep for surgeons.

## References

[1] Yeung AT. Spinal endoscopy with a multichannel, continuous irrigation discoscope with integrated inflow and outflow ports. Poster presentation. Fourth International Meeting on Advanced Spine Techniques, Bermuda. July10–13, 1997.

[2] Yeung AT (1999). Minimally invasive disc surgery with the Yeung Endoscopic SpineSystem (YESS). Surg Tech Int.

[3] Yeung AT (2000). The evolution of percutaneous spinal endoscopy and discectomy: state of the art. Mt Sinai J Med.

[4] Yeung AT (2000). The practice of minimally invasive spinal technique.

[5] Yeung AT, Tsou PM (2002). Posterolateral endoscopic excision for lumbar discherniation. Surgical technique, outcome and complications in 307 consecutive cases. Spine.

[6] Hoogland T, Scheckenbach C (1995). Die perkutane lumbale nukleotomie mit lowdosis chymopapain, ein ambulantes Verfahren. Z Orthop Ihre Grenzgeb.

[7] Hoogland T, Scheckenbach C, Dekkers H. Endoskopische transforaminale diskektomie. Ambulant operieren. 1999:4

[8] Hoogland T, Scheckenbach C (1999). Endoskopische transforaminale fiskektomie (ETD)–Ergebnisse nach 2 Jahren. Orthopädische Praxis.

[9] Hoogland T. Transforaminal endoscopic discectomy with foraminoplasty for lumbar disc herniation. In: Surgical techniques in orthopaedics and traumatology. Paris: Elsevier SAS; 2003:55-120-C-40.

[10] Hoogland T, Schubert M, Miklitz B, Ramirez A (2006). Transforaminal posterolateral endoscopic discectomy with or without the combination of a low-dose chymopapain: a prospective randomized study in 280 consecutive cases. Spine.

[11] Hoogland T, Brekel-Dijkstra KVD, Schubert M, Miklitz B (2008). Endoscopic transforaminal discectomy for recurrent lumbar disc herniation. A prospective, cohort evaluation of 262 consecutive cases. Spine.

[12] MacNab I (1971). Negative disc exploration: an analysis of the causes of nerve root involvement in sixty-eight patients. J Bone Joint Surg Am.

[13] Nakamura SI, Myers RR (2000). Injury to dorsal root ganglia alters innervation of spinal cord dorsal horn lamina involved in nociception. Spine.

[14] Morgan-Hough CVJ, Jones PW, Eisenstein SM (2003). Primary and revision lumbar discectomy. J Bone Joint Surg (Br).

